# Personality traits and sexual self‐efficacy in diabetic women: The mediating role of marital satisfaction and sexual function

**DOI:** 10.1002/brb3.2371

**Published:** 2021-09-23

**Authors:** Masoumeh Jabbari, Monirolsadate Hosseini ‐Tabaghdehi, Zahra Kashi, Nouraddin Mousavinasab, Zohreh Shahhosseini

**Affiliations:** ^1^ Student Research Committee School of Nursing and Midwifery Mazandaran University of Medical Sciences Sari Iran; ^2^ Department of Midwifery Health Reproductive Research Center Sari Branch Islamic Azad University Sari Iran; ^3^ Diabetes Research Center Mazandaran University of Medical Sciences Sari Iran; ^4^ Health Sciences Research Center Mazandaran University of Medical Sciences Sari Iran; ^5^ Sexual and Reproductive Health Research Center Mazandaran University of Medical Sciences Sari Iran

**Keywords:** diabetes, marital satisfaction, personality, sexual function, sexual self‐efficacy, women

## Abstract

**Introduction:**

Although the relationship between personality traits and sexual self‐efficacy has received theoretical and empirical support, there is little information on how personality affects the sexual self‐efficacy of diabetic women. This study aimed to investigate the mediating role of marital satisfaction and sexual function in the relationship between personality traits and sexual self‐efficacy in diabetic women.

**Methods:**

Using a two‐stage sampling method, 410 reproductive‐aged Iranian women with type 2 diabetes were recruited in this descriptive‐analytical study. The participants completed self‐administered questionnaires, including the Vaziri Sexual Self‐Efficacy Questionnaire, the Female Sexual Function Index, ENRICH Marital Satisfaction Scale, and the Goldberg's Big Five Questionnaire. To analyze the data, structural equation modeling was employed in Amos software version 24.

**Results:**

Results indicated an acceptable fit of the model to the data. Personality trait of openness to experience was associated with sexual self‐efficacy directly (*β* = .02, *p *= .030) and indirectly through the mediators of marital satisfaction (*β* = .06, *p *= .009) and sexual function (*β* = .18, *p *= .014). Furthermore, sexual function was associated with sexual self‐efficacy directly (*β* = .50, *p *= .025) and indirectly through the mediator of marital satisfaction (*β* = .36, *p* = .012).

**Conclusion:**

Due to the mediating role of marital satisfaction and sexual function, this study has some practical implications for improving the sexual self‐efficacy of diabetic women with different personality traits.

## Abstract 2

The relationship between personality traits and sexual self‐efficacy has received theoretical and empirical support, although there is little information on how personality affects the sexual self‐efficacy of diabetic women.

## 1 INTRODUCTION

Diabetes is one of the most common non‐communicable chronic diseases of the century, and the global prevalence of this disease has been estimated to increase from 9.3% in 2019 to 10.2% in 2030 (Saeedi et al., 2019). More than three million people have diabetes (type 1 and 2) in Iran; given its latent type, 20% of the population has been affected by or is susceptible to diabetes. The number of diabetic patients in Iran will probably reach more than nine million by 2030 (Javanbakht et al., 2015).

Some studies have reported a prevalence of sexual dysfunction in diabetic women between 17 and 94/4% (Rahmanian et al., 2019) that occurs due to physiological and psychological changes (Maiorino et al., 2014). This may affect the individuals' sexual life, including their sexual self‐efficacy (Assarzadeh et al., 2019; Bailes et al., 2013; Steinke et al., 2008; Tehrani et al., 2014; Vaziri et al., 2010).

Sexual self‐efficacy refers to one's belief in an effective and appropriate sexual function in his/her sexual partner and the ability to achieve her/his sexual satisfaction (Lou et al., 2011). Closson et al. (2018) stated that this factor controls a person's sexual life and the ability to engage in safe sexual behaviors. Hence, several studies have shown that young people with higher sexual self‐efficacy have less high‐risk sexual activity (Rosenthal et al., 1991). Furthermore, Steinke et al. (2008) emphasized that sexual self‐efficacy can lead to a desirable sexual relationship, healthy sexual activities, and sexual and general health promotion. Therefore, improving sexual self‐efficacy, regarding the related variables, is a substantial component in promoting healthy sexual behaviors (Lou et al., 2011; Powwattana & Ramasoota, 2008; Reissing et al., 2005).

Theoretical and empirical findings have supported the relationship between personality traits and sexual self‐efficacy (Crisp et al., 2015; Firoozi et al., 2016; Schaffhuser et al., 2014), and as Çolakoğlu and Gözükara (2016) clarify, personality traits are constructs for explaining behavioral patterns in each individual's personal life. When people with different personality traits marry, their marital relationships and their sexual life can be affected by their personality traits (Sadeghi et al., 2016).

Due to the high prevalence of sexual dysfunction in reproductive‐aged women with type 2 diabetes (Rahmanian et al., 2019), and the relationship between sexual self‐efficacy and sexual dysfunction, investigating the long‐lasting relevant factors affecting a person's sexual life in different societies is necessary to improve women's sexual self‐efficacy. Besides, the importance of women's sexual health highlights the need for a strategy for early diagnosis of the problem and the application of possible interventions in diabetic women to support psychological adjustment and prevent the complications of emotional disorders. A lot of studies have shown the mediating role of marital satisfaction in various areas related to health, including the couple's relationship, attachment styles, and mental health (Chung & Choi, 2014; Peplińska et al., 2013). Few studies have been conducted to investigate how personality traits affect the sexual self‐efficacy of women with diabetes. The present study was designed to determine the path analysis fit of the conceptual model, which measures the mediating role of marital satisfaction and sexual function in the relationship between personality traits and sexual self‐efficacy in diabetic women.

## 2 MATERIALS AND METHODS

In this cross‐sectional descriptive‐analytical study, 410 reproductive‐aged women with type 2 diabetes, ranging in age from 15 to 49, participated between May and September 2019.

### 2.1 Sampling

According to the study conducted by Ghasemi et al. (2020), the mean and standard deviation of self‐efficacy scores were 16.74 and 7.07, respectively. Then, the sample size was calculated using the following formula:

(1) $$n\;=\frac{\sigma^{2}*{\left(z_{1-\frac{\alpha }{2}}+z_{1-\beta }\right)}^{2}}{{\left(\mu_{1}-\mu_{0}\right)}^{2}}$$

Given that the confidence level was 95% and the power was 80%, the calculation revealed that 410 participants were required for this study. The authors administered a two‐stage sampling method to select the samples. Based on the population of the diabetic patients in the two endocrine centers affiliated with Mazandaran University of Medical Sciences in the north of Iran, the proportion of samples in each center was calculated. Thus, 260 samples were assigned to the first clinic and 150 to the second one. Afterward, the eligible patients were selected from the daily list of the physician's visits and were informed about the research goals. In this step, sampling was done through simple random sampling based on a random number table (selecting a maximum of two participants per day at each center). After completing the written informed consent, the participants filled out the self‐administered questionnaires, too.

### 2.2 Inclusion and exclusion criteria

Inclusion criteria comprised of married literate women with type 2 diabetes (at least one year after the diagnosis) aged between 15 and 49 years who were willing to participate in the study. Exclusion criteria encompassed pregnant, menopause, and lactating women, as well as those with advanced diabetes (who had experienced amputation and vision, kidney, and heart problems). Finally, women with a history of hospitalization in psychiatric wards were also omitted from the study.

‐ The demographic‐socio‐medical information form included questions on age, education, occupation of the couples, age of marriage, body mass index, duration of diabetes, comorbidities, and medication use.
‐ The Vaziri Sexual Self‐Efficacy Questionnaire contains ten questions with two domains, namely, factor 1 (items 1–6) and factor 2 (items 7–10). It is scored based on 4‐choice items ranging from 0 (not correct) to 3 (completely correct). The total score ranges from zero to 30; higher scores indicate higher sexual self‐efficacy. Vaziri and Lotfi Kashani (2013) confirmed the reliability of the questionnaire using Cronbach's alpha (*α* = .86), and the Spearman‐Brown split‐half method (0.81). In the present study, Cronbach's alpha of the questionnaire in a population of 20 diabetic women was .77, and the Spearman correlation coefficient in a 2‐week interval was .96.
‐ Rosen et al. (2000) introduced the Female Sexual Function Index that measures women's sexual function in six domains with 19 items, namely, desire (items 1–2), arousal (items 3–6), lubrication (items 7–10), orgasm (items 11–13), satisfaction (items 14–16), and pain (items 17–19). Scoring is based on the five‐point Likert scale, with scores of 1–5 or 0–5 for each domain. The maximum score for each domain is six, and for the whole scale is 36; the higher the score, the better the sexual function. The reliability of the Persian version of the questionnaire was confirmed by Cronbach's alpha coefficient of .70 and a correlation coefficient of .85 (Heydari & Faghihzadeh, 2008).
‐ The ENRICH Marital Satisfaction Scale consists of 35 questions in four domains, marital satisfaction (10 items), communication (10 items), conflict resolution (10 items), and ideal distortion (5 items). Scoring is based on the five‐point Likert scale, ranging from strongly agree (score 1) to strongly disagree (score 5). The range of the scores varies between 35 and 165, with higher scores indicating greater marital satisfaction (Asoodeh et al., 2010; Rostami & Gol, 2014). Cronbach's alpha coefficient of .78 and correlation coefficient of .90 confirmed the reliability of the questionnaire (Daneshpour et al., 2011)
‐ The 50‐item Goldberg's Big Five Questionnaire with 5 scales is used to assess and evaluate five personality traits in individuals. The five components include emotional stability, extroversion, openness to experience, agreeableness, and conscientiousness. Items are based on a five‐point Likert scale ranging from very false for me (score 1) to very true for me (score 5); the higher score among the five components indicates the predominant personality trait in the individual. The Cronbach's alpha coefficient of the components of this tool varies between .77 and .88 (Goldberg, 1999; Khormaei, 2007).

### 2.3 Ethical considerations

First, we got the ethics code from Mazandaran University of Medical Sciences (IR.MAZUMS.REC.1398.6026). Then, we obtained informed written consent from the participants and explained the research project and its objectives. They were also assured about the confidentiality of the collected data.

### 2.4 Statistical analysis

Employing SPSS version 18, we calculated the mean and standard deviation to depict the quantitative variables, while frequency and percentage were used for describing the qualitative variables. Moreover, Amos version 24 was employed to analyze the relationship between the variables. The main variables studied in the model were sexual self‐efficacy as the dependent variable, marital satisfaction and sexual function as the mediating variables, and personality traits as the independent variable. The basis of data analysis was the structural equation modeling (SEM) with a significance level of .05 in all tests. In this way, bias‐corrected bootstrap, a two‐way test showing the significance of the direct and indirect effects between variables, was employed to report the 95% confidence interval along with its lower and upper limit for the mediator variables in SEM. If zero is not observed in this interval, an indirect effect of the mediating variable is confirmed.

## 3 RESULTS

In this study, the participants filled in the questionnaires while waiting at the endocrine centers and six women did not complete them due to lack of time (response rate = 98.5%). The mean ages of the participants and their spouses in this study were 41.81 ± 6.88 and 47.14 ± 9.31 years, respectively. Moreover, the mean score and standard deviation of the marriage age and duration were 20.18 ± 5.14 and 21.56 ± 9.05 years. Finally, the mean and standard deviation of the diabetes duration was 7.6 ± 92.4 years. Most of the participants were high school dropouts (46.60%) and housewives (79.80%), with two children (41.20%).

**FIGURE 1:**
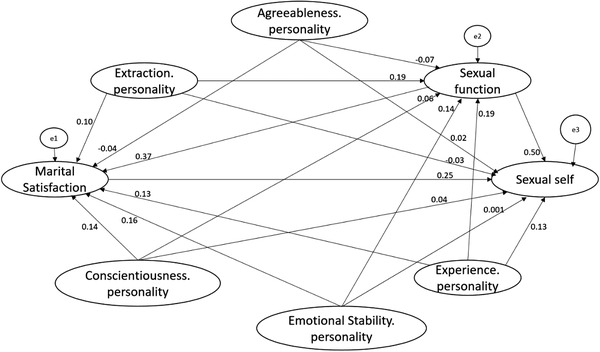
The final research measurement model and its parameters using standardized data

Descriptive statistics (i.e., mean, standard deviation, and correlation between the variables) are shown in Table 1. As illustrated, there is a significant correlation between the variables at the .01 and .05 significance levels. The major purpose of the research measurement model was to determine whether the structural model of the mediating role of marital satisfaction and sexual function enjoys an appropriate fit concerning personality traits and sexual self‐efficacy relationships in diabetic women. The initial model was examined by structural equations using maximum likelihood estimation. In the final model, the quantity of $\chi^{2}/\mathrm{df}$ was equal to 2.01, the comparative fit index (CFI) was .90, the parsimony comparative fit index (PCFI) was .84, and the root mean square error of approximation (RMSEA) was .05 (the accessibility cut points for each model fit indexes: $\chi^{2}/\mathrm{df}$ < 5, CFI > .9, PCFI > .5 and RMSEA < .05), indicating the acceptability of the goodness of fit model index (Schreiber et al., 2006). Figure 1 demonstrates the research final measurement model and its parameters, and Table 2 displays the parameters of the research measurement model using SEM.

**TABLE 1:** The descriptive statistics for independent, mediator, and dependent variables among reproductive‐aged diabetic women

	Descriptive statistics		Correlations among variables							
Variables	Mean	SD	1	2	3	4	5	6	7	8
1. Marital satisfaction	118.71	1.14	1							
2. Sexual function	22.81	9.06	.431**	1						
3. Extroversion	25.93	.28	.237**	.250**	1					
4. Agreeableness	34.28	.39	.143**	.101*	.361**	1				
5. Conscientiousness	42.42	.27	.224**	.231**	.233**	.338**	1			
6. Emotional stability	41.97	.26	.297**	.311**	.176**	.194**	.402**	1		
7. Openness to experience	30.85	.44	.299**	.259**	.357**	.347**	.441**	.381**	1	
8. Sexual self‐efficacy	33.24	.39	.531**	.624**	.162**	.129**	.283**	.299**	.357	1

* Correlation is significant at the .05 level (two‐tailed).

** Correlation is significant at the .01 level (two‐tailed).

**TABLE 2:** Parameters of the research measurement model using SEM

	*b*	SE	*β*	*p*‐value
Extroversion→Sexual function	.21	.06	.21	<.0001
Agreeableness→Sexual function	–.08	.06	–.10	.192
Conscientiousness→Sexual function	.08	.08	.07	.337
Emotional stability→Sexual function	.11	.04	.16	.006
Openness to experience→Sexual function	.16	.05	.18	<.0001
Extroversion→Marital satisfaction	.09	.05	.08	.054
Agreeableness→Marital satisfaction	–.04	.05	−.03	.441
Conscientiousness→Marital Satisfaction	.16	.06	.13	.014
Emotional stability→Marital satisfaction	.11	.03	.16	.001
Openness to experience→Marital satisfaction	.10	.04	.11	.010
Sexual function→Marital satisfaction	.31	.05	.36	<.0001
Marital satisfaction→Sexual self‐efficacy	.20	.04	.26	<.0001
Extroversion→Sexual self‐efficacy	–.02	.03	–.02	.524
Agreeableness→Sexual self‐efficacy	.02	.03	.01	.610
Conscientiousness→Sexual self‐efficacy	.04	.04	.06	.367
Emotional stability→Sexual self‐efficacy	.001	.02	.02	.979
Openness to experience→Sexual self‐efficacy	.08	.03	.14	.004
Sexual function→Sexual self‐efficacy	.34	.04	.50	<.0001

As can be observed in Table 2, the highest standardized regression coefficient exists between sexual function and sexual self‐efficacy (*β* = .50, *p *< .0001), sexual function and marital satisfaction (*β* = .36, *p *< .0001), and marital satisfaction and sexual self‐efficacy (*β* = .26, *p *< .0001), consecutively.

The results reported in Table 3 display the direct and indirect standardized effects of the variables and the significance level of this correlation based on the bias‐corrected percentile method test. The results showed that the “openness to experience” personality trait has a statistically significant relationship with sexual self‐efficacy, directly (*β* = .02, *p *= .030) and indirectly through the mediators of marital satisfaction (*β* = .06, *p* = .009) and sexual function (*β* = .18, *p* = .014). Other personality traits did not have any direct relationship with sexual self‐efficacy, but the “extroversion” personality trait was indirectly associated with sexual self‐efficacy through the mediating role of sexual function (*β* = .19, *p* = .032) and marital satisfaction (*β* = .06, *p *= .029). Moreover, emotional stability was indirectly correlated with sexual self‐efficacy through mediating role of sexual function (*β* = .14, *p *= .016) and marital satisfaction (*β* = .16, *p *= .029). Sexual function was directly (*β* = .50, *p *= .025) and indirectly (*β* = .36, *p *= .012) associated with sexual self‐efficacy through the mediating role of sexual satisfaction. Furthermore, a significant direct relationship was observed between marital satisfaction and sexual self‐efficacy (*β* = .25, *p *= .0005).

**TABLE 3:** Direct and indirect standardized effects of variables in the final research model

	Extroversion		Agreeableness		Conscientiousness		Emotional stability		Openness to experience		Sexual function		Marital satisfaction	
	D	I	D	I	D	I	D	I	D	I	D	I	D	I
Sexual function	.19*		–.07		.05		.14*		.18*					
Marital satisfaction	.10	.06*	–.04	–.02	.14	.01	.16*	.04*	.13	.06**	**.36** *			
Sexual self‐efficacy	–.03	.14*	.02	–.05	.04	.06	.00	.12**	.02*	.24	**.50** *	**.08** **	**.25** **	

D, direct effect; I, indirect effect. The blank cells indicated that there is no reported effect at analysis.

* *p*‐value < .05.

** *p*‐value < .01.

## 4 DISCUSSION

Sexual self‐efficacy, as a fundamental component of sexual health, is influenced by various personal and interpersonal variables in different socio‐cultural contexts (Potki et al., 2017). Based on the results of the structural equation model, the personality trait of “openness to experience” was associated with sexual self‐efficacy directly, as well as indirectly through the mediators of marital satisfaction and sexual function. Furthermore, sexual function was associated with sexual self‐efficacy directly, and indirectly through the mediator of marital satisfaction. In other words, marital satisfaction and sexual function act as mediating variables in the relationship between personality traits and sexual self‐efficacy of diabetic reproductive‐aged women. This issue can expand our knowledge of the sexual self‐efficacy of these women.

Ryckman (2012) claims that personality is a psychological or structural concept composed of unique characteristics influencing one's thoughts, emotions, and behaviors in different situations. There are several models for interpreting and explaining personality. In this study, researchers used a five‐factor model based on SEM to determine the relationship between sexual self‐efficacy and personality traits. This model includes five dimensions: extroversion, agreeableness, conscientiousness, emotional stability, and openness to experience (Mullins‐Sweatt & Lengel, 2012; Rector et al., 2012). The present study demonstrated that the “openness to experience” has a significant direct and indirect correlation with sexual self‐efficacy of diabetic reproductive‐aged women through sexual function and marital satisfaction. This personality factor brings about an increase in the desire to pay attention to the talks and wishes of the spouse and to understand his views. In general, increasing the sense of aesthetics, diversity seeking, curiosity, accepting new ideas, and flexibility enhance marital satisfaction. However, McCrae and Costa (2008) believe that “openness to experience” is the most complicated factor among the five main personality traits, and it is difficult to understand and detect it.

This study also revealed that “extroversion” and “emotional stability” were indirectly associated with sexual self‐efficacy through marital satisfaction and sexual function. Since extroversion is associated with positive emotions such as intimacy, optimism, happiness, pleasure, and love, it can create a robust marital relationship between the couples, which increases marital satisfaction (Karney & Bradbury, 1995). However, Alikamali et al. (2017) do not confirm this finding.

In this study, no significant relationship was found between the other dimensions of personality traits (agreeableness and conscientiousness) and sexual self‐efficacy. Thus, further studies are required to accurately and clearly understand this issue.

The authors found that sexual function was directly and indirectly associated with sexual self‐efficacy with the mediating role of marital satisfaction in the present research. Since the sexual instinct is a human need and one of the main reasons for marriage, the quality of a sexual relationship may influence marital satisfaction. Although a happy marriage is only partially concerned with sex, dissatisfaction in a marital relationship can lead to deprivation, frustration, and family breakdown. The sexual relationship is one of the key relationships in couples, and it is believed that there is an association between sexual and marital satisfaction. Therefore, any factor that can affect sexual satisfaction and improve the relationship can also be helpful in marital satisfaction (Pedro et al., 2015).

Similar to previous studies, this study reported a significant positive correlation between marital satisfaction and sexual function with sexual self‐efficacy (Kafaei Atrian et al., 2019; Vaziri et al., 2010; Zimmer‐Gembeck, 2013). According to the World Health Organization (2010), sexual function is an essential part of women's sexual health; therefore, health centers providing sexual health services should consider promoting, enhancing, and maintaining sexual function, too. Some studies have found that sexual self‐efficacy training increases marital satisfaction by increasing sexual satisfaction (Hughes et al., 2006; Shokrani, 2020). Furthermore, Reissing et al. (2005) concluded that optimal sexual self‐efficacy is concerned with sexual compatibility and increased sexual activity. Sexual satisfaction is a person's pleasant feeling in a sexual relationship. In addition, high levels of sexual satisfaction can enhance the quality of life and increase marital stability. On the other hand, the concept of marital satisfaction, as sexual satisfaction or emotional satisfaction, guarantees an understanding of self‐efficacy. Therefore, sexual self‐efficacy can also play an important role in marital satisfaction (Bakhshayesh & Mortazavi, 2010; Steinke et al., 2008; Vaziri et al., 2010).

### 4.1 Strengths and limitations

One of the strengths of the present study is that data analysis with SEM can show the simultaneous effect of several independent variables on the dependent variable. This model can process latent variables that are not directly observed; it is a measurement model that is more flexible than other statistical methods. Another strength of the present study is the sufficient and acceptable number of samples that randomly entered the study.

However, similar to any other study, our study is subject to some limitations. First, due to the cross‐sectional nature of this study, it is impossible to determine the cause‐and‐effect relationships between sexual self‐efficacy, sexual function, and marital satisfaction of the participants because of temporality bias. Second, information bias is conceivable because of the sensitivity of sexual health questions, especially in the conservative society context in developing countries where cultural constraints may prevent disclosing the relationship between couples. To reduce this bias, we reassured the participants that the data were confidential. However, the results may be prone to social desirability bias. Finally, the results of this study cannot be generalized to women with other types of diabetes.

Therefore, it is recommended that further studies be conducted on women with other types of diabetes to compare the results with those of the present study. Experimental studies are also recommended to work on the mediating factors among these patients and to improve the sexual self‐efficacy of diabetic women through instruction.

## 5 CONCLUSION

Improving sexual self‐efficacy is an appropriate strategy to prevent sexual problems in reproductive‐aged diabetic women. Therefore, it is important to pay attention to the role of major effective and mediating factors in formulating these strategies. The present study confirmed the mediating role of marital satisfaction and sexual function in the relationship between different personality traits and sexual self‐efficacy in diabetic women. Therefore, it is recommended that actions be taken to provide the women with low sexual self‐efficacy with the necessary information about the effect of personality traits on sexual self‐efficacy. Besides, strategies should be employed aiming at sexual satisfaction and function.

## CONFLICT OF INTEREST

The authors declare that there is no conflict of interest.

## AUTHOR CONTRIBUTIONS

Masoumeh Jabbari and Zohreh Shahhosseini contributed to the design of the study. Masoumeh Jabbari, Zohreh Shahhosseini, Monirolsadate Hosseini Tabaghdehi, and Nouraddin Mousavinasab contributed to the implementation and analysis plan. Zahra Kashi and Masoumeh Jabbari contributed to data collection. Monirolsadate Hosseini Tabaghdehi and Zohreh Shahhosseini wrote the first draft of this manuscript.

### PEER REVIEW

The peer review history for this article is available at https://publons.com/publon/10.1002/brb3.2371.

## Data Availability

The data that support the findings of this study are available from the corresponding author, upon reasonable request.
